# Molecular Characterization and Functional Analysis of a Putative Octopamine/Tyramine Receptor during the Developmental Stages of the Pacific Oyster, *Crassostrea gigas*

**DOI:** 10.1371/journal.pone.0168574

**Published:** 2016-12-16

**Authors:** Peng Ji, Fei Xu, Baoyu Huang, Yingxiang Li, Li Li, Guofan Zhang

**Affiliations:** 1 Key Laboratory of Experimental Marine Biology, Institute of Oceanology, Chinese Academy of Sciences, Qingdao, Shandong, China; 2 University of Chinese Academy of Sciences, Beijing, China; 3 National & Local Joint Engineering Laboratory of Ecological Mariculture, Institute of Oceanology, Chinese Academy of Sciences, Qingdao, Shandong, China; 4 Laboratory for Marine Biology and Biotechnology, Qingdao National Laboratory for Marine Science and Technology, Qingdao, Shandong, China; 5 Laboratory for Marine Fisheries and Aquaculture, Qingdao National Laboratory for Marine Science and Technology, Qingdao, Shandong, China; University of Hong Kong, HONG KONG

## Abstract

Octopamine (OA) and its precursor, tyramine (TA), participate in invertebrate development such as growth, maturation, and reproduction by activating their corresponding G protein-coupled receptors (GPCRs). Although OA was first discovered in mollusks (octopus), subsequent studies on OA, TA and related receptors have primarily been conducted in Ecdysozoa, especially in insects. Accordingly, only limited reports on OA/TA receptors in mollusks are available and their physiological roles remain unclear. Here, a full-length cDNA encoding a putative 524 amino acid OA/TA receptor (CgGPR1) was isolated from the Pacific oyster *Crassostrea gigas*. CgGPR1 was most closely related to the *Lymnaea stagnalis* OA receptor OAR2 in sequence. Phylogenetic analysis showed that CgGPR1 belongs to a poorly studied subfamily of invertebrate OA/TA receptors. The spatio-temporal expression of CgGPR1 in *C*. *gigas* larvae was examined by quantitative real-time PCR and Western blot analysis. CgGPR1 was expressed during all developmental stages of *C*. *gigas* with higher levels at mid-developmental stages, indicating its potential role in embryogenesis and tissue differentiation. Immunoreactive fluorescence of CgGPR1 was mainly observed in the velum, foot, gill and mantle of *C*. *gigas* larvae. *CgGPR1* transcripts were detected in all the tested organs of adult *C*. *gigas*, with highest level in the mantle. Pharmacological analysis showed that cAMP and Ca^2+^ concentrations remained unchanged in HEK293 cells expressing CgGPR1 upon addition of OA, TA or related amines, suggesting that CgGPR1 modulates other unknown molecules rather than cAMP and Ca^2+^. Our study sheds light on CgGPR1 function in oysters.

## Introduction

Biogenic amines, including octopamine (OA), tyramine (TA), and other amines, comprise a large family of basic neuroactive factors and have significant physiological functions in most organisms ranging from microorganisms to animals. OA, first discovered in mollusks (octopus), is synthesized from the amino acid tyrosine through TA in the animal body. Previous studies on OA and TA have primarily been focused on Ecdysozoa. In insects, OA represents one of the major biogenic amines and plays important roles in aggressive behaviors, sleep and reproductive processes. While in vertebrates, similar functions are usually associated with both adrenaline and noradrenaline [1–3]. TA, as the precursor of OA, also performs many functional roles in invertebrates, including olfaction in insects and reversal behavior in *Caenorhabditis elegans* [4, 5]. However, little is known regarding OA and TA in mollusks.

The function of biogenic amines is mediated by G protein-coupled receptors (GPCRs), which are considered as the largest family of cell-surface receptors. GPCRs share significant levels of sequence homology and common features, including seven hydrophobic transmembrane domains (7TM) linked by six hydrophilic loops, an extracellular amino-terminus, and an intracellular carboxy-terminus [6]. Via the binding of ligands, GPCRs are able to activate intracellular heterotrimeric G proteins and then further activate downstream effectors, along with effecting changes in the levels of second messengers such as cAMP, Ca^2+^, inositol triphosphate and diacylglycerol (DAG). In this manner, extracellular signals can be transduced to the interior of the cell. Cellular signal transduction occurs mainly through two second-messenger pathways, the adenylate cyclase/cyclic AMP (AC/cAMP) pathway and the phosphatidylinositol/diacylglycerol/protein kinase C (PI/DAG/PKC) pathway [7, 8]. GPCRs have been shown to function in many developmental stages of various marine invertebrate species through either or both of these signal transduction pathways [9–12].

OA receptors, together with other biogenic amine receptors such as TA, dopamine (DA), adrenergic and noradrenergic receptors, belong to the rhodopsin class of GPCRs (GPCRdb, http://gpcrdb.org), which has been extensively studied owing to their crucial functions in vertebrates. While TA-OA and DA-noradrenaline represent tyrosine derivatives synthesized by two different pathways, their receptors exhibit close homology. Accordingly, the invertebrate OA/TA receptors have recently been classified into four subtypes, α_1_-adrenergic-like OA receptors, α_2_-adrenergic-like TA receptors, β-adrenergic-like OA receptors and OA/TA receptors with unknown effectors, according to the classification and pharmacological properties of the corresponding vertebrate adrenergic receptors [13, 14].

OA receptors have been found to be involved in a broad range of developmental events, such as the metamorphosis of *Drosophila* [15], the maturation of sexual behavior in the male moth *Agrotis ipsilon* [16], and the processing of sensory inputs, antennal motor outputs, and higher-order brain functions in the honeybee *Apis mellifera* [17]. TA receptors, on the other hand, were reported to play important roles in the olfactory system of the moth *Heliothis virescens* [18], brain development of the honeybee *A*. *mellifera* [19] and larval locomotion of the fruit fly *Drosophila melanogaster* [20]. Although the function of OA/TA receptors has been well studied in insects, reports on the role of these receptors in mollusks are quite limited. Previous studies have identified some octopamine receptors in several mollusks such as *Spisula solidissima*, *Lymnaea stagnalis*, and *Crassostrea virginica* [14, 21–23]. These studies proposed that OA/TA receptors could play crucial roles in some physiological activities of mollusks such as reproductive viability and nervous actions. However, the signal transduction mediated by these receptors and their expression patterns and related physiological roles in the development of mollusks remain to be studied. Thus, it is necessary to further study OA/TA receptors in mollusks.

The Pacific oyster *Crassostrea gigas* (Thunberg 1793) represents a well-known species of mollusk, which has recently gained considerable attention for its importance in aquaculture and marine ecosystems. Based on the genome sequences and expression profile data of the Pacific oyster released in 2012 [24], we identified and annotated 553 GPCR genes. Among these, one bioamine receptor gene exhibited specifically high expression levels during mid-developmental and metamorphic stages, suggesting its particular roles in oyster development. In this study, we cloned and characterized this novel bioamine receptor gene with respect to its phylogenetic relationships and spatio-temporal expression patterns in the development of *C*. *gigas*. In addition, in vitro functional analysis was performed to reveal its potential role in biogenic amine-mediated intracellular signaling.

## Materials and Methods

### Ethics Statement

The oysters used in this study were obtained from local mariculture farms and cultured in the aquarium at Institute of Oceanology, Chinese Academy of Sciences (IOCAS). All of the experiments from this research were designed and conducted according to the regulations of the local and central governments. No specific permissions were required for the oyster sample collection and other experiments described in this study. All of the field studies were carried out at IOCAS, and did not involve any endangered or protected species.

### Sample collection and treatment

Larvae and organ samples of the Pacific oyster *C*. *gigas* used in this study were collected from an aquaculture farm in Qingdao, China. The embryo and larvae samples at different developmental stages were identified microscopically and collected using a screen mesh, including the egg (E), two cells (TC), four cells (FC), early morula (EM), morula (M), blastula (B), gastrula (G), trochophore (T), early D-shaped larva (ED), D-shaped larva (D1, D2, and D3), early umbo larva (EU), umbo larva (U1, U2, and U3), pediveliger larva (P), and spat (S) stages. Adult organs including the hemocyte, mantle, gill, labial palp, adductor muscle, gonad, and digestive gland were dissected from three individual oysters.

Samples used for whole-mount immunofluorescence assays were collected and treated as described previously [25]. In brief, larvae samples from different developmental stages of *C*. *gigas* were directly fixed in fresh 4% paraformaldehyde in 0.01 M phosphate buffered saline (PBS) for 2 h at 4°C and washed three times (15 min each) with cold PBS. Starting from the D-shaped larvae stage, samples were relaxed by gradual addition of 7.5% MgCl_2_ prior to fixation. After fixation, the samples were immediately transferred into 70% ethanol and stored at −20°C for subsequent immunofluorescence assessment.

### RNA isolation and cDNA synthesis

Total RNA from each sample was extracted using TRIzol Reagent (Invitrogen, Carlsbad, CA, USA) following the manufacturer’s protocol, then immediately treated with DNase I (Promega, Madison, WI, USA) to remove genomic DNA contamination. The concentration and quality of the RNA samples were measured by a NanoDrop 2000 spectrophotometer (Thermo Scientific, Waltham, MA, USA) and 1.0% agarose gel electrophoresis. Subsequently, first-strand cDNA was synthesized using the PrimeScript RT Reagent Kit with gDNA Eraser (TaKaRa, Shiga, Japan) according to the manufacturer’s protocol.

### Molecular cloning of the novel bioamine receptor gene *CgGPR1*

The complete coding sequences (CDS) of *CgGPR1* were amplified by PCR based on the bioinformatics prediction of the gene model of CGI_10017568 using cDNA from oyster gills as the template. To acquire the complete cDNA sequence of *CgGPR1*, 5′ and 3′ rapid amplification of cDNA ends (RACE) procedures were performed as previously reported [26]. Primer sequences used in the amplification of *CgGPR1* are listed in the supplementary material (S1 Table). All of the PCR products of expected size were excised and purified using the GenElute Gel Extraction kit (Sigma-Aldrich, St. Louis, MO, USA), subcloned into the pMD-19T simple vector (TaKaRa), and sequenced in both directions (Sangon Biotech, Shanghai, China).

### Sequence analysis and phylogenetic tree construction

The nucleotide and deduced amino acid sequences of CgGPR1 were analyzed and compared using the BLAST program at the National Center for Biotechnology Information (http://blast.ncbi.nlm.nih.gov/Blast.cgi). The transmembrane segments and topology of CgGPR1 were predicted by TMHMM 2.0 (http://www.cbs.dtu.dk/services/TMHMM-2.0/). The NetPhos 2.0 Server (http://www.cbs.dtu.dk/services/NetPhos/) and NetNGlyc 1.0 Server (http://www.cbs.dtu.dk/services/NetNGlyc/) were used to analyze the phosphorylation and glycosylation sites of CgGPR1, respectively. Multiple protein sequence alignment was carried out using the MUSCLE program. The phylogenetic tree was constructed using the MEGA v.6.0 software with the neighbor joining method.

### Quantification of *CgGPR1* expression

Quantitative real-time PCR (qPCR) was performed on the ABI 7500 Fast Real-Time PCR System (Applied Biosystems, CA, USA) using the SYBR Green real-time PCR mix (TaKaRa). The qPCR was carried out in a total volume of 20 μl, containing 10 μl 2× SYBR Premix Ex Taq, 1 μl diluted cDNA, 0.4 μl each of the forward and reverse primers, 0.4 μl 50× ROX Reference Dye II, and 7.8 μl DEPC-treated water. The elongation factor (*EF*) gene and the ribosomal protein S18 (*RS18*) gene were used as internal controls to normalize the mRNA expression of *CgGPR1* in different organs and developmental stages, respectively [27, 28]. All of the reactions were conducted in triplicate within 96-well optical reaction plates (Applied Biosystems). The conditions of the qPCR program were 95°C for 2 min, followed by 40 cycles of 95°C for 3 s and 60°C for 30 s. Melting curve testing was performed to confirm the specificity of the amplifications and the comparative Ct method (2^−ΔΔCt^ method) was used to analyze the mRNA expression level of the target genes [29].

### Western blotting analysis

To assess the protein expression pattern of CgGPR1, a polyclonal rabbit anti-CgGPR1 antibody was synthesized by Abmart, Inc. (Shanghai, China). A single peptide (MIPNLYKFNIETKR) was selected from CgGPR1 and then produced by chemosynthesis for the production of a polyclonal antibody in rabbits. The specificity of the synthetic polyclonal antibody was verified by enzyme-linked immunosorbent assay (ELISA) according to previous protocol [30]. Subsequently, Western blotting analysis of CgGPR1 was conducted as described previously [31] with slight modification. Briefly, larvae samples from different developmental stages of *C*. *gigas* were first sonicated on ice in Radio Immunoprecipitation Assay lysis buffer; the lysates were then clarified by centrifugation at 4°C (10,000×g for 5 min). The supernatant was collected and then incubated at 100°C for 5 min. Subsequently, extracted proteins were separated on 12.5% sodium dodecyl sulfate gels and transferred onto a nitrocellulose membrane. The membrane was blocked for 2 h with 5% (w/v) non-fat dry milk in TBS buffer containing 0.1% Tween-20 (TBST) and incubated for 2 h with the anti-CgGPR1 polyclonal antibody at a 1:50 dilution in TBST. Then the membrane was washed 3 times (15 min each) with TBST and incubated with the secondary antibody (horseradish peroxidase-conjugated goat anti-rabbit IgG antibody, ABclonal Technology, Cambridge, MA, USA) at a 1:10,000 dilution in TBST. After washing with TBST 4 times for 15 min each, the membrane was treated with a chemiluminescence kit (Millipore, Billerica, MA, USA) and chemiluminescent signals were visualized by exposure to X-ray films. In addition, Western blotting analysis of β-actin using a rabbit anti-β-actin IgG antibody (ABclonal Technology) was also performed as an internal control to normalize the protein expression level of CgGPR1 in different developmental stages.

### Whole-mount immunofluorescence assay

Whole-mount immunofluorescence assay was performed as described previously [25]. Briefly, larvae samples from different developmental stages of *C*. *gigas* were washed in 0.01 M PBS 3 times for 15 min each. The shells of larvae (D-shaped larvae and later) were decalcified with 5% EDTA solution in PBS for 30 min at room temperature, followed by washing 3 times for 15 min each with PBS. Afterwards, the specimens were blocked at 4°C overnight in blocking solution (10% normal goat serum, 0.25% bovine serum albumin, 1% Triton X-100, and 0.03% sodium azide in PBS) and then incubated with the rabbit anti-CgGPR1 antibody (1:50 dilution in blocking solution) at 4°C for 3 days. Subsequently, these specimens were washed 3 times for 20 min each with PBST (PBS buffer containing 0.05% Tween-20) and incubated with the goat anti-rabbit IgG antibody conjugated to Alexa Fluor 488 (1:600 dilution in blocking solution; Invitrogen) for 1 day at room temperature. The specimens were then washed 5 times for 15 min each with PBST and mounted in 80% glycerol in PBS for subsequent detection. The negative control was established by incubating the specimens with preimmune sera plus secondary antibody. Finally, all of the specimens were examined and analyzed as whole-mount using the Zeiss Laser-Scanning Confocal Microscopy System LSM 710 (Zeiss, Jena, Germany).

### Stable expression and pharmacological characterization of CgGPR1

The complete coding sequence of *CgGPR1* was subcloned into the Lenti-puro vector and packaged by lentivirus, followed by the stable transfection of human embryonic kidney (HEK293) cells (ATCC, Manassas, VA, USA). HEK293 cells were cultured as described previously [14]. Stably expressing polyclonal cell lines were screened and constructed by incubation in the presence of puromycin for several weeks and the expression level of CgGPR1 was measured and verified with qPCR and fluorescence-activated cell sorting (FACS). By using the limited dilution method, a single monoclonal cell line that stably expressed CgGPR1 was selected for further study. This monoclonal cell line was then seeded in a 384-well plate (black-wall, clear-bottom) at a suitable density in plating medium, and maintained in 5% CO_2_ at 37°C, followed by incubation for 30 min at room temperature. The levels of cAMP and Ca^2+^ were subsequently measured and analyzed using the Cyclic AMP Assay Kit (Cisbio, Bedford, MA, USA) and FLIPR Calcium Assay Kit (Molecular Devices, Sunnyvale, CA, USA), respectively. Samples were treated according to the manufacturer's protocols and various concentrations of solution ranging from 10 μM to 50 mM for 6 different biogenic amines (OA, TA, DA, L-DOPA, adrenaline, and noradrenaline) were added into the corresponding wells of the assay plate at the appropriate time.

The values obtained in this experiment are represented as the mean ± standard deviation (SD) of triplicate independent experiments. The tests of normality (Shapiro-Wilk test) and homogeneity test of variance (F-test) showed that most of these values followed a normal distribution and homogeneity of variance. We thus used one-way analysis of variance (ANOVA) to test the difference, followed by a multiple comparison. For data failed to pass the normality test, non-parametric test (Mann-Whitney test) was used to determine the statistical significance. All the analysis were conducted using the SPSS software package (v. 13.0; Chicago, IL, USA). Differences were considered statistically significant at *P* < 0.05 and extremely significant at *P* < 0.01.

## Results and Discussion

### Cloning and sequence analysis of CgGPR1 in *C*. *gigas*

Via PCR and RACE-PCR-based methods, a full length cDNA encoding a putative G-protein-coupled receptor was cloned from the gill of *C*. *gigas*, which was named CgGPR1 (GenBank Accession No. KX710104). The identified cDNA sequence was 2,826 bp in length including a 762-bp 5′-untranslated region (UTR), a 1,575-bp open reading frame (ORF), and a 489-bp 3′-UTR with a polyA tail (S1 Fig). The ORF encodes a putative protein of 524 amino acid residues with a calculated molecular weight of 60 kDa. Comparison between the cDNA sequence of CgGPR1 and the genomic sequence of *C*. *gigas* [24] suggested that no intron is present in the coding region of the gene. The gene model is consistent with the bioinformatics prediction with the exception of a 12 bp difference at the 5′UTR and a 17 bp difference at the 3′UTR.

**Fig 1:**
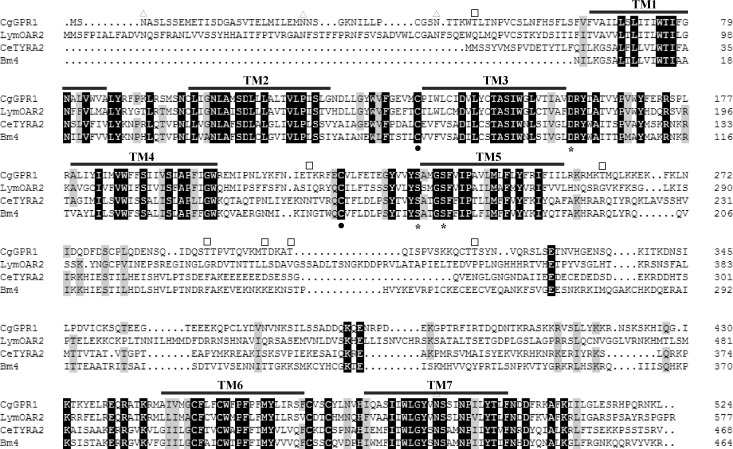
Multiple sequence alignment of CgGPR1 and orthologous receptors. The alignment was performed by ClustalX. The predicted seven transmembrane regions (TM1-TM7) are overlined. Identical and similar amino acids are shaded in black and gray, respectively. Potential N-glycosylation sites and phosphorylation sites for protein kinase C are indicated by triangles and squares, respectively. Amino acid residues that are implicated in agonist binding are labeled by asterisks. Conserved Cys residues in the first and second extracellular loops are indicated by filled circles.

The predicted CgGPR1 amino acid sequence exhibits the characteristic features of the GPCR family [32]. Hydrophobicity analysis revealed that CgGPR1 contains seven putative transmembrane segments (TM) connected by intra and extra-cellular loops including an extracellular N-terminus and a cytoplasmic C-terminus, and that CgGPR1 and its homologs have a relatively large loop connecting the TM5 and TM6 regions (Fig 1). These transmembrane regions harbor residues involved in biogenic amine binding and are conserved between CgGPR1 and its closely related receptors (Fig 1). In CgGPR1, the D_159_ residue in the conserved D-R-F motif downstream of TM3 and the Ser residues (S_232_ and S_236_) in the conserved S-x-x-x-S motif located in TM5 are predicted to be involved in agonist binding [33]. CgGPR1 also contains a conserved L_104_-x-x-x-D motif located in TM2 and a conserved N_493_-P-x-x-Y in TM7, which are thought to be characteristics of catecholaminergic receptors and involved in ligand-induced internalization [34]. Two highly conserved Cys residues (C_135_ and C_220_) are also found in the first and second extracellular loops of CgGPR1, which are considered to stabilize the protein structure by formation of a disulfide bridge [35]. Notably, both CgGPR1 and the mollusk *L*. *stagnalis* octopamine receptor OAR2 (LymOAR2) lack the second conserved Phe residue following the F-x-x-x-W-x-P motif located at the middle of TM6. In CgGPR1, this Phe residue is replaced by Pro and in LymOAR2 by Leu (Fig 1). CgGPR1 contains four potential sites for N-linked glycosylation, three (N_3_, N_28_, and N_41_) in the extracellular N-terminal region and one (N_489_) in TM7 (Fig 1). In addition, seven Thr residues were identified as potential sites for phosphorylation by protein kinase C (Fig 1).

BLAST analysis against the NCBI non-redundant protein database (nr) revealed that CgGPR1 shows the most significant sequence identity with characterized LymOAR2 (42%) [22], the *Caenorhabditis elegans* TA receptor TYRA2 (32%) [36], and the *Brugia malayi* TA receptor Bm4 (29%) [37], suggesting that CgGPR1 is likely to be a biogenic amine receptor.

### CgGPR1 is a member of the invertebrate OA/TA receptor family

**Fig 2:**
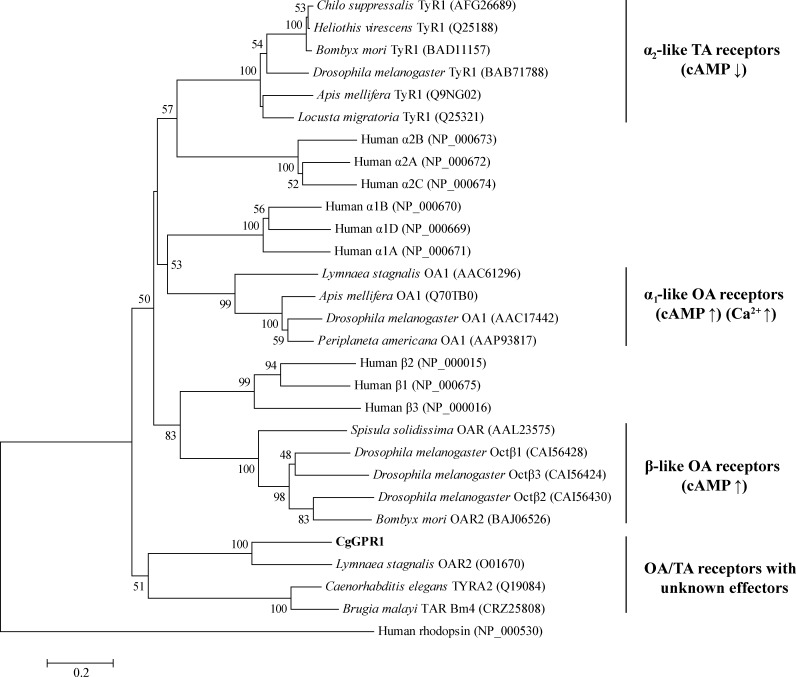
Phylogenetic tree of CgGPR1 and various biogenic amine receptors. The tree was built by the Neighbor Joining method with a JTT matrix-based model using 259 amino acid positions. Bootstrap analysis of 1000 replicates was conducted and values above 50% are shown. The scale for branch length is shown below the tree. Human rhodopsin receptor (NP_000530) was used as an outgroup.

A phylogenetic tree was constructed to investigate the relationship between CgGPR1 and other biogenic amine receptors from mollusks, other invertebrates and mammals (Fig 2). As shown in Fig 2, the CgGPR1 protein belongs to the cluster of the invertebrate OA/TA receptor family. As previously described, the family of invertebrate OA/TA receptors are classified into four subfamilies based on similarities in structure and signaling properties with vertebrate adrenergic receptors: (1) α-adrenergic-like OA receptors, (2) β-adrenergic-like OA receptors, (3) octopamine/tyramine or tyramine receptors (also called α_2_-adrenergic-like receptors), and (4) octopamine/tyramine receptors with unknown effectors [13, 14]. CgGPR1 clustered within the fourth subfamily of the invertebrate OA/TA receptor family (Fig 2). Compared to the extensive research performed with respect to the former three subfamilies, the study on the fourth subfamily of invertebrate OA/TA receptors is quite limited. To date, this subfamily contains only three known members, *L*. *stagnalis* OAR2 [22], *C*. *elegans* TYRA2 [36] and *B*. *malayi* Bm4 [37]. Among these, CgGPR1 was phylogenetically most closely related to *L*. *stagnalis* OAR2 (Fig 2), which is consistent with the sequence homology search results.

### CgGPR1 expression levels during different developmental stages of *C*. *gigas*

**Fig 3:**
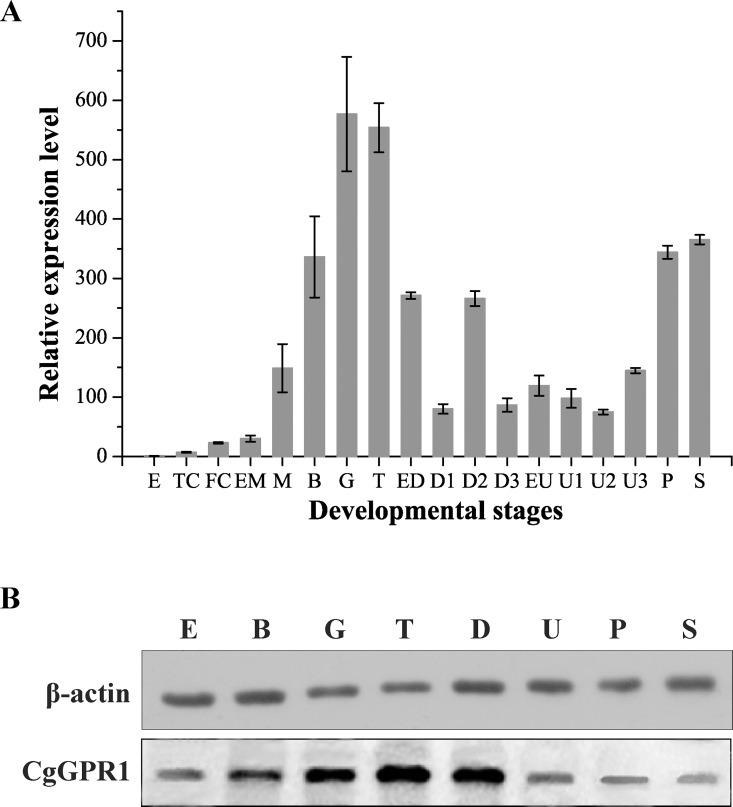
Expression patterns of CgGPR1 in the developmental stages of *C*. *gigas*. (A) Expression patterns of *CgGPR1* transcripts. *RS18* gene was used as the internal control. Data are represented as the mean ± SD of triplicate independent experiments. (B) Analysis of CgGPR1 in *C*. *gigas* larvae by Western blot using anti-CgGPR1 antibodies. β-actin was used as the loading control. E, egg; TC, two cells; FC, four cells; EM, early morula; M, morula; B, blastula; G, gastrula; T, trochophore; ED, early D-shaped larva; D, D-shaped larva; EU, early umbo larva; U, umbo larva; P, pediveliger; S, spat.

qPCR was performed to determine the distribution of *CgGPR1* transcripts in different developmental stages of *C*. *gigas*. The mRNA transcripts of *CgGPR1* were expressed at a relatively high level during the whole developmental stages except for the egg stage (Fig 3A). The level of *CgGPR1* transcripts was the lowest in egg and increased gradually in the following development stages, reaching its highest value in gastrula and trochophore approximately 560-fold higher than that in egg. Subsequently, the expression decreased and was maintained at a moderate level from D-larva to umbo larva, whereupon it increased again in pediveliger and spat. It has been accepted that tissue differentiation and organ formation usually occur after the gastrula stage during ontogenesis in mollusks [38]. The high expression of *CgGPR1* in the mid-developmental stages indicated that this receptor is likely involved in the embryogenesis, tissue differentiation and organ formation of *C*. *gigas*. Furthermore, the significant increase of *CgGPR1* expression in pediveliger after umbo larva suggested that it might also be involved in *C*. *gigas* metamorphosis.

To further confirm the expression pattern of CgGPR1 in *C*. *gigas*, the distribution of CgGPR1 protein in different developmental stages was assayed by Western blot. CgGPR1 protein was detected in all the developmental stages from egg to spat with high levels in gastrula, trochophore and D-shaped larva (Fig 3B), supporting that CgGPR1 is likely to play a role in the embryogenesis and tissue differentiation of *C*. *gigas*. Notably, the expression pattern of CgGPR1 protein is consistent with its mRNA expression pattern (Fig 3A).

### Spatial expression of CgGPR1 during the larval development of *C*. *gigas*

**Fig 4:**
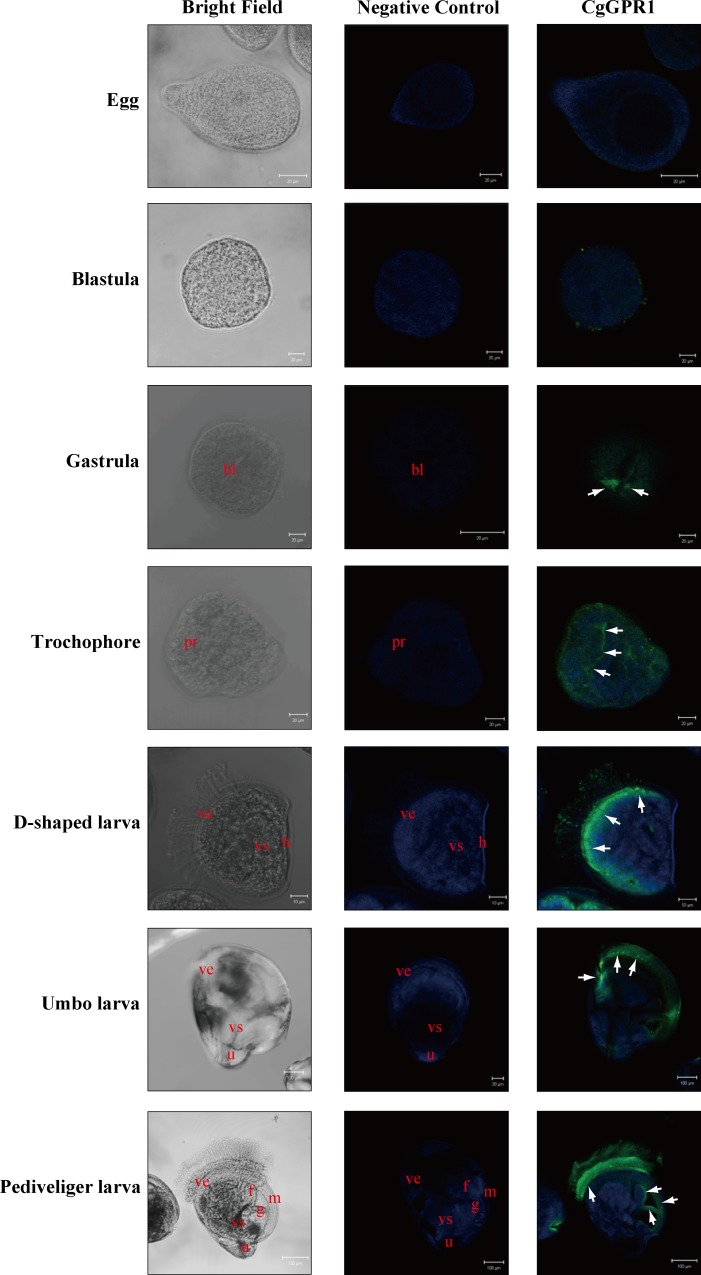
Whole-mount immunofluorescence of CgGPR1 in *C*. *gigas* larvae. The immunoreactive area was marked with arrow. bl, blastopore; pr, prototroch; ve, velum; vs, visceral mass; h, hinge; u, umbo; f, foot; g, gill; m, mantle.

The whole-mount immunofluorescence technique [39] was used to further study the role of CgGPR1 in the organ development of *C*. *gigas* larvae. The immunoreactivity of CgGPR1 was first observed near the blastopore of gastrula and then in the prototroch of trochophore (Fig 4). At the developmental stages from D-shaped larva to pediveliger, strong immunopositive fluorescence of CgGPR1 was mainly detected in the velum, especially in the base of the cilia. Some immunopositive fluorescence was also observed in the foot, gill and mantle of pediveliger. No CgGPR1 immunoreactive fluorescence was found in the negative controls of the seven larval stages (Fig 4).

CgGPR1 was expressed in the prototroch of trochophore (Fig 4). Upon its development into the velum in early D-shaped larvae, CgGPR1 was also expressed therein (Fig 4). Molluscan larvae commonly use ciliated vela to swim and feed [40]. In addition to the cilia, the velar musculature also plays important roles in locomotion and feeding [41]. It has been documented that catecholamines affect the activity of cilia and muscles in the velum and the swimming behavior of intact larvae of *Ilyanassa obsoleta* under neural control [42]. At the metamorphic stages (pediveliger and spat), the velum in bivalves is gradually degenerated [43]. As shown in Fig 4, CgGPR1 was present in the velum during its entire developmental period in *C*. *gigas* larvae, suggesting that CgGPR1 plays important roles in the mid-development of *C*. *gigas*, especially for the velum. At the pediveliger stage of oyster larvae, foot is well-developed and used for locomotion, sensation and attachment [44]. The localization of ganglion in the foot makes oyster sensitive to food, adhering substrates and other components in surrounding environments [45]. Once appropriate substrate is sensed, oyster will settle and undergo a complex process of metamorphosis. After metamorphosis, the foot will be lost [46]. The detection of CgGPR1 on the surface of foot at the pediveliger stage suggested its potential role in *C*. *gigas* attachment and metamorphosis. Gills of bivalve mollusks function as both respiratory and feeding organs. Molluscan gills are rudimentary in D-shaped larvae and become well-developed after metamorphosis [43]. The detection of CgGPR1 in the gill at the pediveliger stage of *C*. *gigas* suggested that CgGPR1 is also important for the development of oyster gills. Mantle is the main shell secreting organ in bivalve mollusks [47]. The detection of CgGPR1 in the mantle of *C*. *gigas* larvae implies that CgGPR1 might also be involved in the formation of shell.

### Expression level of CgGPR1 in different organs of adult *C*. *gigas*

**Fig 5:**
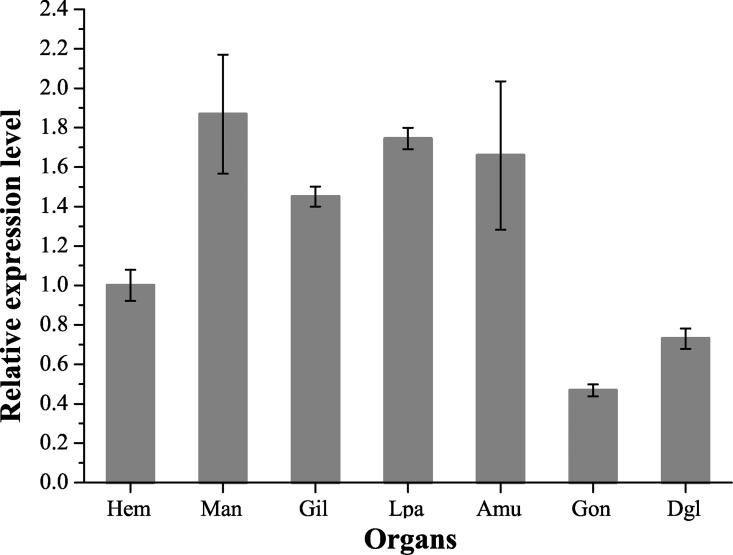
Expression patterns of *CgGPR1* transcripts in different organs of adult *C*. *gigas*. *EF* gene was used as the internal control. Data are represented as the mean ± SD of triplicate independent experiments. Hem, hemocyte; Man, mantle; Gil, gill; Lpa, labial palps; Amu, adductor muscle; Gon, gonad; Dgl, digestive gland.

Via qPCR, *CgGPR1* mRNA expression levels were also determined in different organs of adult *C*. *gigas*. The expression of *CgGPR1* transcripts was detected in all the tested organs including the hemocytes, mantle, gill, labial palps, adductor muscle, gonad, and digestive gland (Fig 5). The highest expression was observed in the mantle and the least in the gonad. The expression of *CgGPR1* transcripts in the mantle was 3-fold higher than that in the gonad. Relatively high expression levels of *CgGPR1* transcripts were also observed in the gill, labial palps and adductor muscle. These results suggested that CgGPR1 is also likely to play roles in adult organs. The expression pattern of CgGPR1 in the organs differed from those of other reported oyster biogenic amine receptors including the adrenergic receptor AR_cga_ [48] and the DA receptor Ca-DA1R [26], which both exhibited highest expression levels in the labial palps. The different expression patterns between CgGPR1 and other tyrosine derivative receptors suggested the functional differentiation of GPCRs for different biogenic amine receptors in oysters. While mantle is one of the main sensing organs, labial palps also function as channels for filtrating and selecting food from the sea water. The high transcript levels of CgGPR1 in mantle and labial palps suggest that it may play important roles in sensory detection.

### Stable expression and pharmacological characterization of CgGPR1

**Fig 6:**
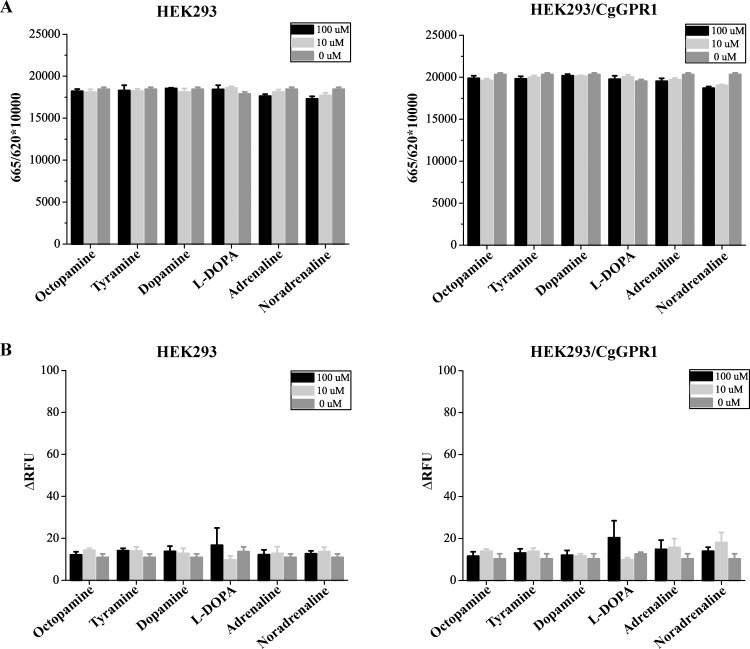
**Effects of various biogenic amines on cAMP (A) and Ca**^**2+**^ **(B) levels in HEK293 cells expressing CgGPR1.** HEK293 cells with (right panels) or without CgGPR1 (left panels) were treated with indicated biogenic amines at different concentrations. cAMP levels were indicated as the ration between the absorbance of the reaction system at 665 nm and 620 nm. Ca^2+^ levels were indicated by the fluorescent intensity of the reaction system (ΔRFU). Treated HEK293 cells without CgGPR1 by biogenic amines and untreated HEK293 cells with CgGPR1 was used as negative controls. Compared to the control groups, no significant changes in cAMP (A) and Ca^2+^ (B) levels were observed in HEK293 cells expressing CgGPR1 upon the addition of octopamine, tyramine and related biogenic amines.

To further investigate the functional properties of CgGPR1, we generated a HEK293 monoclonal cell line that stably expressed CgGPR1 confirmed by both qPCR and FACS (S2 Fig). As phylogenetic analysis indicated that CgGPR1 is a member of the invertebrate OA/TA receptor family (Fig 2) and most OA/TA receptors are reported to cause changes in intracellular cAMP and/or Ca^2+^ concentrations upon ligand binding [14, 49], we then studied the effects of CgGPR1 activation by OA/TA and related amines on these intracellular second messengers. Six biogenic amines were tested including OA, TA, DA, L-DOPA, adrenaline and noradrenaline. However, when assayed at concentrations of 10 or 100 μM, none of the tested amines altered the levels of cAMP and Ca^2+^ in HEK293 cells expressing CgGPR1 (Fig 6A and 6B). Even though the concentrations of amines were increased up to 1 and 50 mM, no significant differences were observed between the experimental groups and the control groups either. This result for cAMP is consistent with previous findings for other homologs within this subfamily [22, 36]. Octopaminergic stimulation of the *L*. *stagnalis* OAR2 did not induce changes in intracellular concentrations of cAMP or inositol phosphates [22]. Activation of the *C*. *elegans* TYRA2 by TA also had no effect on cAMP levels [36]. Although the impact of the *B*. *malayi* Bm4 on cAMP has not been studied, Bm4 may exhibit similar pharmacological characteristics as *C*. *elegans* TYRA2 owing to their high sequence identity (78%) [37]. CgGPR1, together with *L*. *stagnalis* OAR2, *C*. *elegans* TYRA2 and *B*. *malayi* Bm4, are the only reported members of the fourth invertebrate OA/TA receptor subfamily (Fig 2). Therefore, it seems that receptors of this subfamily, unlike other invertebrate OA/TA receptors, modulate molecules other than cAMP, Ca^2+^ and inositol phosphates.

Evidence for potential effectors of the fourth invertebrate OA/TA receptor subfamily might be suggested by several previous studies. Electrophysiological analysis showed that OA was able to activate Cl^−^ efflux of HEK293 cells expressing the *L*. *stagnalis* OAR2 in a manner dependent on protein phosphorylation by unknown protein kinases excluding protein kinase A or C [22]. However, the OA-gated chloride channel in *L*. *stagnalis* modulated by OAR2 remains unidentified. Recently, TA-gated chloride channels were detected in *C*. *elegans* [50] and *Haemonchus contortus* [51]. The mutation of the TA-gated chloride channel protein LGC-55 in *C*. *elegans* blocked the TA-induced suppression of head oscillations and stimulation of backward locomotion, whereas the mutation of the TA receptor TYRA2 had no such effects [50], suggesting that TYRA2 might act on other TA-gated chloride channels or have other physiological roles in *C*. *elegans*. In addition, *C*. *elegans* TYRA2 was found to potentially couple with the G protein subunit Gα_i/o_ [36]. Phylogenetic analysis also suggested that members of the fourth invertebrate OA/TA receptor subfamily were Gα_i/o_-coupled receptors [37], implying that CgGPR1 might also couple with Gα_i/o_. Notably, no vertebrate homologs exist for members of this subfamily, indicating a unique invertebrate-specific evolution for these receptors [14].

Although the CgGPR1 signaling pathways remain unknown, our study on the spatio-temporal expression pattern of CgGPR1 in *C*. *gigas* suggests the important roles for this receptor in the mid-development of *C*. *gigas*, especially for the development of the velum. Additional studies are required to further illustrate the function of CgGPR1. Gene knockdown by transgenic techniques or RNAi will help to verify the function and potential signaling pathways related to CgGRP1, through comparison of the phenotype of *C*. *gigas* larvae in the mid-developmental stage and the expression levels of related genes before and after receptor mutation.

## Conclusions

We cloned the complete cDNA sequence of a putative OA/TA receptor, CgGPR1, from the Pacific oyster *C*. *gigas*. Phylogenetic analysis showed that CgGPR1 belongs to a poorly studied invertebrate OA/TA receptor subfamily that, until now, contained only three known members. Notably, this subfamily has no vertebrate homologs, indicating a unique invertebrate-specific evolution for these receptors. The expression pattern of CgGPR1 in different developmental stages of *C*. *gigas* was examined at both the mRNA and protein level. CgGPR1 was expressed in all developmental stages from egg to spat, with high levels at gastrula, trochophore and D-shaped larva stages, suggesting that CgGPR1 may be involved in embryogenesis and tissue differentiation of *C*. *gigas*. Furthermore, whole-mount immunofluorescence assay was performed to reveal the spatio-temporal expression of CgGPR1 in *C*. *gigas* larvae. Immunoreactive fluorescence of CgGPR1 was first observed near the blastopore of gastrula, then in the prototroch of trochophore and mainly in the velum, foot, gill and mantle in the following developmental stages, suggesting that CgGPR1 plays important roles in the mid-development of *C*. *gigas*, especially for the velum. The expression of *CgGPR1* mRNA was also detected in all the tested organs including the hemocytes, mantle, gill, labial palps, adductor muscle, gonad and digestive gland, implying that CgGPR1 is also required in adult oyster organs. To reveal its physiological role, CgGPR1 was stably expressed in HEK293 cells. The addition of OA/TA and related amines caused no changes in the intracellular levels of cAMP and Ca^2+^ in HEK293 cells expressing CgGPR1. Our pharmacological result is consistent with previous reports for the other members of this subfamily, demonstrating that additional, as yet unknown molecules rather than cAMP and Ca^2+^ may be involved in the regulatory pathways of CgGPR1. In summary, our study provides an insight into the functions of CgGPR1 and its close orthologs in invertebrates.

## Supporting Information

S1 Fig The full-length cDNA sequence and deduced amino acid sequence of CgGPR1. The complete cDNA of CgGPR1 was 2,826 bp in length, including a 762-bp 5’-untranslated region (UTR), a 1,575-bp open reading frame (ORF), and a 489-bp 3’-UTR with a polyA tail.
(TIF)

S2 Fig Verification for the stable expression of CgGPR1 in HEK293 cells. (A) qPCR analysis for the stable expression of CgGPR1 in HEK293 cells. Column 1 represents HEK293 cells without CgGPR1 as the negative control. Columns 2, 3 and 4 represent HEK293 cells with CgGPR1. Data are represented as the mean ± SD of triplicate independent experiments and statistically extremely significant differences compared with the negative control (**P < 0.01). (B) Fluorescence-activated cell sorting (FACS) assay for the stable expression of CgGPR1 in HEK293 cells. The red peak represents HEK293 cells without CgGPR1 as the negative control. The blue peak represents HEK293 cells with CgGPR1.
(TIF)

S1 Table Primer sequences used in the amplification of *CgGPR1*. (DOCX)

## References

[1] Monastirioti M. Distinct octopamine cell population residing in the CNS abdominal ganglion controls ovulation in *Drosophila melanogaster*. Dev Biol. 2003; 264(1):38–49. 14623230 10.1016/j.ydbio.2003.07.019

[2] Crocker A, Sehgal A. Octopamine regulates sleep in *Drosophila* through protein kinase A-dependent mechanisms. J Neurosci. 2008; 28(28):9377–85. 18799671 10.1523/JNEUROSCI.3072-08a.2008 PMC2742176

[3] Hoyer SC, Eckart A, Herrel A, Zars T, Fischer SA, Hardie SL, et al Octopamine in male aggression of *Drosophila*. Curr Biol. 2008; 18(3):159–67. 10.1016/j.cub.2007.12.052 18249112

[4] Kutsukake M, Komatsu A, Yamamoto D, Ishiwa-Chigusa S. A tyramine receptor gene mutation causes a defective olfactory behavior in *Drosophila melanogaster*. Gene. 2000; 245(1):31–42. 10713442 10.1016/s0378-1119(99)00569-7

[5] Alkema MJ, Hunter-Ensor M, Ringstad N, Horvitz HR. Tyramine Functions independently of octopamine in the *Caenorhabditis elegans* nervous system. Neuron. 2005; 46(2):247–60. 10.1016/j.neuron.2005.02.024 15848803

[6] Bockaert J, Pin JP. Molecular tinkering of G protein-coupled receptors: an evolutionary success. EMBO J. 1999; 18(7):1723–9. PubMed Central PMCID: PMC1171258. 10.1093/emboj/18.7.1723 10202136 PMC1171258

[7] Gerhart J. 1998 Warkany lecture: signaling pathways in development. Teratology. 1999; 60(4):226–39. 10.1002/(SICI)1096-9926(199910)60:4<226::AID-TERA7>3.0.CO;2-W 10508976

[8] Holm ER, Nedved BT, Carpizo-Ituarte E, Hadfield MG. Metamorphic-signal transduction in *Hydroides elegans* (Polychaeta: Serpulidae) is not mediated by a G protein. Biol Bull. 1998; 195(1):21–9. 28570195 10.2307/1542772

[9] Pawlik JR. Natural and Artificial Induction of Metamorphosis of Phragmatopoma Lapidosa Californica (*Polychaeta*: *Sabellariidae*), with a Critical Look at the Effects of Bioactive Compounds on Marine Invertebrate Larvae. Bull Mar Sci. 1990; 46(2):512–36.

[10] Clare A, Thomas R, Rittschof D. Evidence for the involvement of cyclic AMP in the pheromonal modulation of barnacle settlement. J Exp Biol. 1995; 198(Pt 3):655–64. 9318389 10.1242/jeb.198.3.655

[11] Yamamoto H, Tachibana A, Matsumura K, Fusetani N. Protein Kinase C (PKC) Signal Transduction System Involved in Larval Metamorphosis of the Barnacle, *Balanus amphitrite*. Zool Sci. 1995; 12(4):391–6.

[12] Amador-Cano G, Carpizo-Ituarte E, Cristino-Jorge D. Role of protein kinase C, G-protein coupled receptors, and calcium flux during metamorphosis of the sea urchin *Strongylocentrotus purpuratus*. Biol Bull. 2006; 210(2):121–31. 10.2307/4134601 16641517

[13] Evans PD, Maqueira B. Insect octopamine receptors: a new classification scheme based on studies of cloned *Drosophila* G-protein coupled receptors. Invert Neurosci. 2005; 5(3–4):111–8. 10.1007/s10158-005-0001-z 16211376

[14] Blais V, Bounif N, Dube F. Characterization of a novel octopamine receptor expressed in the surf clam *Spisula solidissima*. Gen Comp Endocrinol. 2010; 167(2):215–27. 10.1016/j.ygcen.2010.03.008 20302871

[15] Ohhara Y, Shimada-Niwa Y, Niwa R, Kayashima Y, Hayashi Y, Akagi K, et al Autocrine regulation of ecdysone synthesis by beta3-octopamine receptor in the prothoracic gland is essential for *Drosophila* metamorphosis. Proc Natl Acad Sci U S A. 2015; 112(5):1452–7. PubMed Central PMCID: PMC4321272. 10.1073/pnas.1414966112 25605909 PMC4321272

[16] Duportets L, Barrozo RB, Bozzolan F, Gaertner C, Anton S, Gadenne C, et al Cloning of an octopamine/tyramine receptor and plasticity of its expression as a function of adult sexual maturation in the male moth *Agrotis ipsilon*. Insect Mol Biol. 2010; 19(4):489–99. 10.1111/j.1365-2583.2010.01009.x 20491982

[17] Grohmann L, Blenau W, Erber J, Ebert PR, Strunker T, Baumann A. Molecular and functional characterization of an octopamine receptor from honeybee (*Apis mellifera*) brain. J Neurochem. 2003; 86(3):725–35. 12859685 10.1046/j.1471-4159.2003.01876.x

[18] von Nickisch-Rosenegk E, Krieger J, Kubick S, Laage R, Strobel J, Strotmann J, et al Cloning of biogenic amine receptors from moths (*Bombyx mori* and *Heliothis virescens*). Insect Biochem Mol Biol. 1996; 26(8–9):817–27. 9014328 10.1016/s0965-1748(96)00031-8

[19] Mustard JA, Kurshan PT, Hamilton IS, Blenau W, Mercer AR. Developmental expression of a tyramine receptor gene in the brain of the honey bee, *Apis mellifera*. J Comp Neurol. 2005; 483(1):66–75. 10.1002/cne.20420 15672398

[20] Selcho M, Pauls D, El Jundi B, Stocker RF, Thum AS. The role of octopamine and tyramine in *Drosophila* larval locomotion. J Comp Neurol. 2012; 520(16):3764–85. 10.1002/cne.23152 22627970

[21] Gerhardt CC, Bakker RA, Piek GJ, Planta RJ, Vreugdenhil E, Leysen JE, et al Molecular cloning and pharmacological characterization of a molluscan octopamine receptor. Mol Pharmacol. 1997; 51(2):293–300. 9203635 10.1124/mol.51.2.293

[22] Gerhardt CC, Lodder HC, Vincent M, Bakker RA, Planta RJ, Vreugdenhil E, et al Cloning and expression of a complementary DNA encoding a molluscan octopamine receptor that couples to chloride channels in HEK293 cells. J Biol Chem. 1997; 272(10):6201–7. 9045634 10.1074/jbc.272.10.6201

[23] Pryce K, Samuel D, Lagares E, Myrthil M, Bess F, Harris A, et al Presence of Octopamine and an Octopamine Receptor in *Crassostrea virginica*. In Vivo (Brooklyn). 2015; 37(1):16–24. PubMed Central PMCID: PMC4652596. 26594670 PMC4652596

[24] Zhang G, Fang X, Guo X, Li L, Luo R, Xu F, et al The oyster genome reveals stress adaptation and complexity of shell formation. Nature. 2012; 490(7418):49–54. 10.1038/nature11413 22992520

[25] Yue F, Shi X, Zhou Z, Wang L, Wang M, Yang J, et al The expression of immune-related genes during the ontogenesis of scallop *Chlamys farreri* and their response to bacterial challenge. Fish Shellfish Immunol. 2013; 34(3):855–64. 10.1016/j.fsi.2012.12.023 23318996

[26] Yang B, Ni J, Zeng Z, Shi B, You W, Ke C. Cloning and characterization of the dopamine like receptor in the oyster *Crassostrea angulata*: expression during the ovarian cycle. Comp Biochem Physiol B Biochem Mol Biol. 2013; 164(3):168–75. 10.1016/j.cbpb.2012.12.006 23274282

[27] Du Y, Zhang L, Xu F, Huang B, Zhang G, Li L. Validation of housekeeping genes as internal controls for studying gene expression during Pacific oyster (*Crassostrea gigas*) development by quantitative real-time PCR. Fish Shellfish Immunol. 2013; 34(3):939–45. 10.1016/j.fsi.2012.12.007 23357023

[28] Huang W, Xu F, Li J, Li L, Que H, Zhang G. Evolution of a novel nuclear receptor subfamily with emphasis on the member from the Pacific oyster *Crassostrea gigas*. Gene. 2015; 567(2):164–72. 10.1016/j.gene.2015.04.082 25956376

[29] Livak KJ, Schmittgen TD. Analysis of Relative Gene Expression Data Using Real-Time Quantitative PCR and the 2^−ΔΔCT^ Method. Methods. 2001; 25(4):402–8. 10.1006/meth.2001.1262 11846609

[30] Wu T, Shi X, Zhou Z, Wang L, Wang M, Wang L, et al An iodothyronine deiodinase from *Chlamys farreri* and its induced mRNA expression after LPS stimulation. Fish Shellfish Immunol. 2012; 33(2):286–93. 10.1016/j.fsi.2012.05.011 22609768

[31] Chambon JP, Nakayama A, Takamura K, Mcdougall A, Satoh N. ERK- and JNK-signalling regulate gene networks that stimulate metamorphosis and apoptosis in tail tissues of ascidian tadpoles. Development. 2007; 134(6):1203–19. 10.1242/dev.002220 17332536

[32] Strader CD, Fong TM, Graziano MP, Tota MR. The family of G-protein-coupled receptors. FASEB J. 1995; 9(9):745–54. 7601339

[33] Barak LS, Tiberi M, Freedman NJ, Kwatra MM, Lefkowitz RJ, Caron MG. A highly conserved tyrosine residue in G protein-coupled receptors is required for agonist-mediated beta 2-adrenergic receptor sequestration. J Biol Chem. 1994; 269(4):2790–5. 7507928

[34] Kurten RC. Sorting motifs in receptor trafficking. Adv Drug Deliv Rev. 2003; 55(11):1405–19. 14597138 10.1016/j.addr.2003.07.003

[35] Strader CD, Fong TM, Tota MR, Underwood D, Dixon RA. Structure and function of G protein-coupled receptors. Annu Rev Biochem. 1994; 63:101–32. 10.1146/annurev.bi.63.070194.000533 7979235

[36] Rex E, Hapiak V, Hobson R, Smith K, Xiao H, Komuniecki R. TYRA-2 (F01E11.5): a *Caenorhabditis elegans* tyramine receptor expressed in the MC and NSM pharyngeal neurons. J Neurochem. 2005; 94(1):181–91. 10.1111/j.1471-4159.2005.03180.x 15953361

[37] Smith KA, Rex EB, Komuniecki RW. Are *Caenorhabditis elegans* receptors useful targets for drug discovery: pharmacological comparison of tyramine receptors with high identity from *C*. *elegans* (TYRA-2) and *Brugia malayi* (Bm4). Mol Biochem Parasitol. 2007; 154(1):52–61. PubMed Central PMCID: PMC3430142. 10.1016/j.molbiopara.2007.04.004 17537528 PMC3430142

[38] Haszprunar G, Wanninger A. Molluscs. Curr Biol. 2012; 22(13):R510–4. 10.1016/j.cub.2012.05.039 22789994

[39] Dickinson AJ, Croll RP. Development of the larval nervous system of the gastropod *Ilyanassa obsoleta*. J Comp Neurol. 2003; 466(2):197–218. 10.1002/cne.10863 14528448

[40] Mackie GO, Singla CL, Thiriot-Quievreux C. Nervous control of ciliary activity in gastropod larvae. Biol Bull. 1976; 151(1):182–99. 10.2307/1540713 963121

[41] Fretter V. The prosobranch veliger. Proc Malacol Soc London. 1967; 37(6):357–66.

[42] Braubach OR, Dickinson AJ, Evans CC, Croll RP. Neural control of the velum in larvae of the gastropod, *Ilyanassa obsoleta*. J Exp Biol. 2006; 209(Pt 23):4676–89. 10.1242/jeb.02556 17114401

[43] Bayne, B. L. Some morphological changes that occur at the metamorphosis of the larvae of Mytilus edulis. 1971.

[44] Lane DJW, Nott JA. A study of the morphology, fine structure and histochemistry of the foot of the pediveliger of *Mytilus edulis* L. J Mar Biol Assoc UK. 1975; 55(55):477–95.

[45] Croll RP, Dickinson AJG. Form and function of the larval nervous system in molluscs. Invertebr Reprod Dev. 2004; 46(2–3):173–87.

[46] Yang B, Li L, Pu F, You W, Huang H, Ke C. Molecular cloning of two molluscan caspases and gene functional analysis during *Crassostrea angulata* (Fujian oyster) larval metamorphosis. Mol Biol Rep. 2015; 42(5):963–75. 10.1007/s11033-014-3833-y 25399080

[47] Harper EM. The molluscan periostracum: An important constraint in bivalve evolution. Palaeontology. 1997; 40(1):71–97.

[48] Yang B, Qin J, Shi B, Han G, Chen J, Huang H, et al Molecular characterization and functional analysis of adrenergic like receptor during larval metamorphosis in *Crassostrea angulata*. Aquaculture. 2012; 366–367:54–61.

[49] Wu SF, Huang J, Ye GY. Molecular cloning and pharmacological characterisation of a tyramine receptor from the rice stem borer, *Chilo suppressalis* (Walker). Pest Manag Sci. 2013; 69(1):126–34. 10.1002/ps.3378 23129510

[50] Pirri JK, McPherson AD, Donnelly JL, Francis MM, Alkema MJ. A tyramine-gated chloride channel coordinates distinct motor programs of a *Caenorhabditis elegans* escape response. Neuron. 2009; 62(4):526–38. PubMed Central PMCID: PMC2804440. 10.1016/j.neuron.2009.04.013 19477154 PMC2804440

[51] Rao VT, Accardi MV, Siddiqui SZ, Beech RN, Prichard RK, Forrester SG. Characterization of a novel tyramine-gated chloride channel from *Haemonchus contortus*. Mol Biochem Parasitol. 2010; 173(2):64–8. 10.1016/j.molbiopara.2010.05.005 20471431

