# Supplementary figures and images for: Molecular Characterization and Functional Analysis of a Putative Octopamine/Tyramine Receptor during the Developmental Stages of the Pacific Oyster, Crassostrea gigas
Source: PLoS One. 2016 Dec 16;11(12):e0168574. doi: 10.1371/journal.pone.0168574 (PMC5161484; doi:10.1371/journal.pone.0168574)

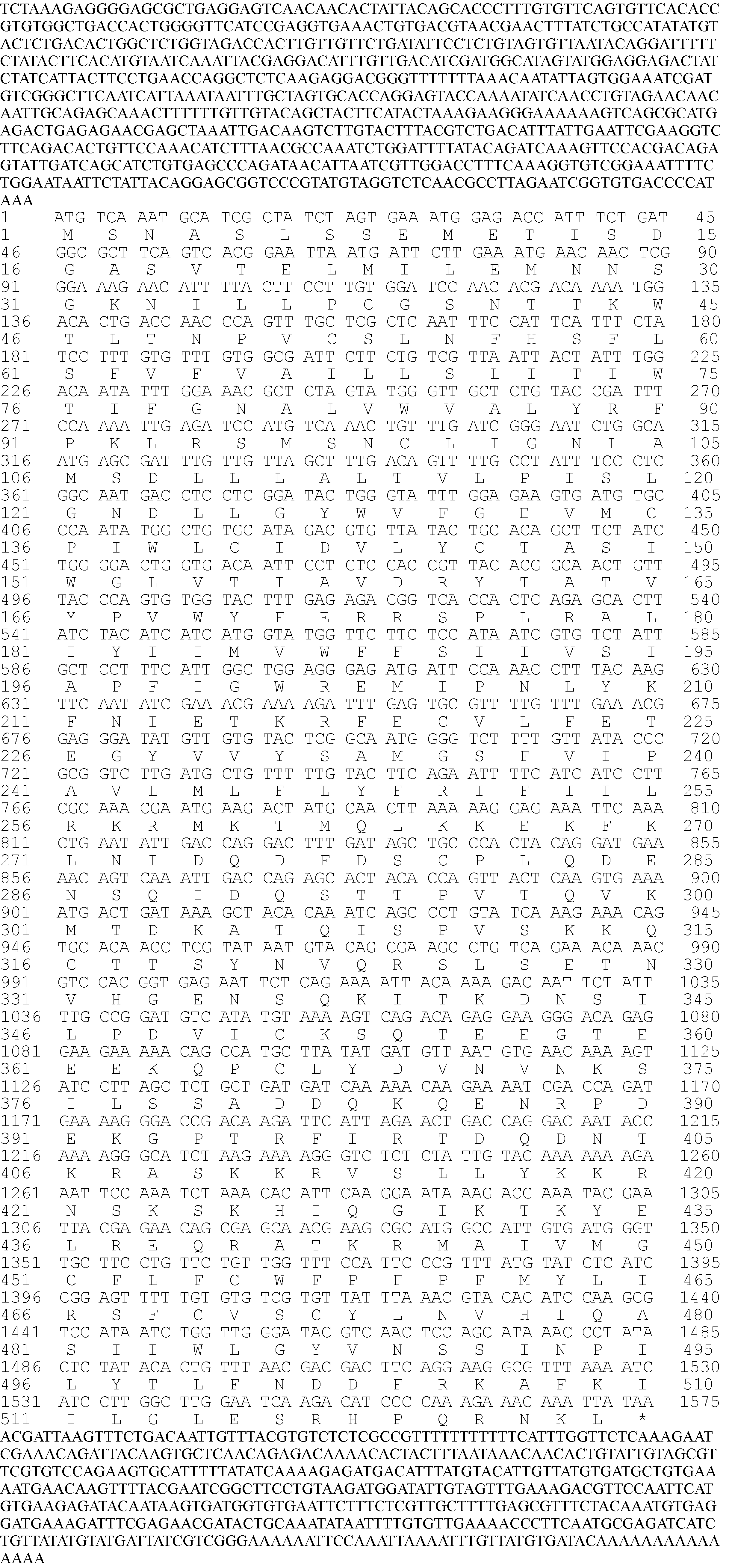

Supplement: S1 Fig — The complete cDNA of CgGPR1 was 2,826 bp in length, including a 762-bp 5’-untranslated region (UTR), a 1,575-bp open reading frame (ORF), and a 489-bp 3’-UTR with a polyA tail. (TIF) [file pone.0168574.s001.tif]

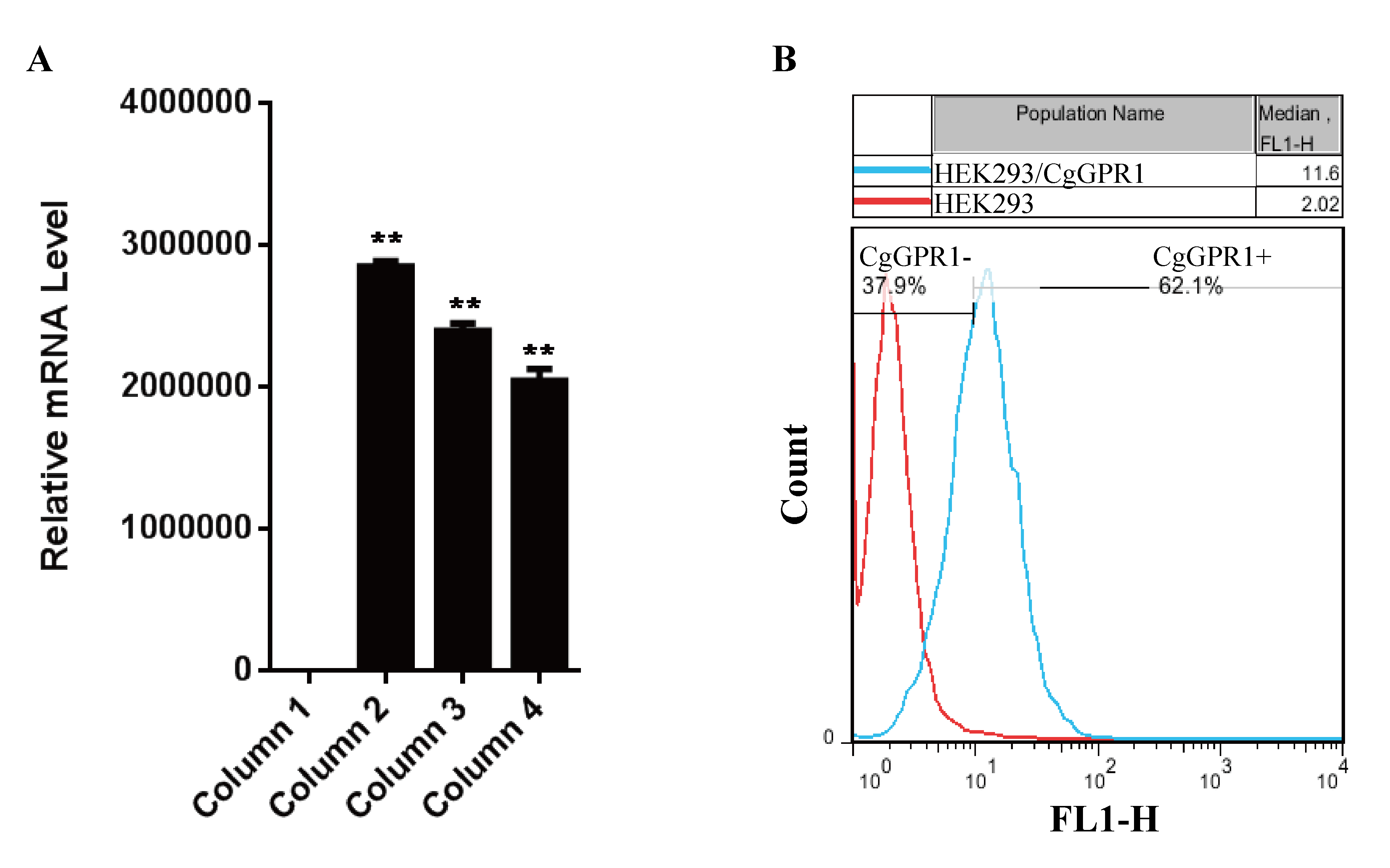

Supplement: S2 Fig — (A) qPCR analysis for the stable expression of CgGPR1 in HEK293 cells. Column 1 represents HEK293 cells without CgGPR1 as the negative control. Columns 2, 3 and 4 represent HEK293 cells with CgGPR1. Data are represented as the mean ± SD of triplicate independent experiments and statistically extremely significant differences compared with the negative control (**P < 0.01). (B) Fluorescence-activated cell sorting (FACS) assay for the stable expression of CgGPR1 in HEK293 cells. The red peak represents HEK293 cells without CgGPR1 as the negative control. The blue peak represents HEK293 cells with CgGPR1. (TIF) [file pone.0168574.s002.tif]
