# Supplementary material for: Molecular Characterization and Functional Analysis of a Putative Octopamine/Tyramine Receptor during the Developmental Stages of the Pacific Oyster, Crassostrea gigas
Source: PLoS One. 2016 Dec 16;11(12):e0168574. doi: 10.1371/journal.pone.0168574 (PMC5161484; doi:10.1371/journal.pone.0168574)
Supplement: S1 Table — (DOCX) [file pone.0168574.s003.docx]

**S1 Table. Primer sequences used in the amplification of *CgGPR1*.**

| **Primers** | **Sequences (5'-3')** | **Application** |
| --- | --- | --- |
| CgGPR1-F | ATGATTCTTGAAATGAACAACTCG | Clone |
| CgGPR1-R | AGTGTATAGAGTATAGGGTTTATGCTGG | Clone |
| CgGPR1F1 | ACCGACAAGATTCATTAGAACTG | 3'-RACE |
| CgGPR1F2 | AGACGAAATACGAATTACGAGAAC | 3'-RACE |
| CgGPR1F3 | GTCAACTCCAGCATAAACCCTA | 3'-RACE |
| dT-adaptor | GGCCACGCGTCGACTAGTACT_16_ | 3'-RACE |
| CgGPR1R1 | CCAGTATCCGAGGAGGTCAT | 5'-RACE |
| CgGPR1R2 | ACGACAGAAGAATCGCCACA | 5'-RACE |
| CgGPR1R3 | TTCTTTCCCGAGTTGTTCAT | 5'-RACE |
| dG-adaptor | GGCCACGCGTCGACTAGTACG_10_ | 5'-RACE |
| qCgGPR1-F | TCGTGTCTATTGCTCCTTTCATTGGC | qPCR |
| qCgGPR1-R | CCGAGTACACAACATATCCCTCCGT | qPCR |
